# Grammatical verb aspect and event roles in sentence processing

**DOI:** 10.1371/journal.pone.0189919

**Published:** 2017-12-29

**Authors:** Carol Madden-Lombardi, Peter Ford Dominey, Jocelyne Ventre-Dominey

**Affiliations:** 1 Université Lyon 1, INSERM U1208, Stem Cell and Brain Research Institute, Integrative Neuroscience Department, Bron, France; 2 Centre National de la Recherche Scientifique, Lyon, France; Max Planck Institute for Human Cognitive and Brain Sciences, GERMANY

## Abstract

Two experiments examine how grammatical verb aspect constrains our understanding of events. According to linguistic theory, an event described in the perfect aspect (John had opened the bottle) should evoke a mental representation of a finished event with focus on the resulting object, whereas an event described in the imperfective aspect (John was opening the bottle) should evoke a representation of the event as ongoing, including all stages of the event, and focusing all entities relevant to the ongoing action (instruments, objects, agents, locations, etc.). To test this idea, participants saw rebus sentences in the perfect and imperfective aspect, presented one word at a time, self-paced. In each sentence, the instrument and the recipient of the action were replaced by pictures (John was using/had used a *corkscrew* to open the *bottle* at the restaurant). Time to process the two images as well as speed and accuracy on sensibility judgments were measured. Although experimental sentences always made sense, half of the object and instrument pictures did not match the temporal constraints of the verb. For instance, in perfect sentences aspect-congruent trials presented an image of the corkscrew closed (no longer in-use) and the wine bottle fully open. The aspect-incongruent yet still sensible versions either replaced the corkscrew with an in-use corkscrew (open, in-hand) or the bottle image with a half-opened bottle. In this case, the participant would still respond “yes”, but with longer expected response times. A three-way interaction among Verb Aspect, Sentence Role, and Temporal Match on image processing times showed that participants were faster to process images that matched rather than mismatched the aspect of the verb, especially for resulting objects in perfect sentences. A second experiment replicated and extended the results to confirm that this was not due to the placement of the object in the sentence. These two experiments extend previous research, showing how verb aspect drives not only the temporal structure of event representation, but also the focus on specific roles of the event. More generally, the findings of visual match during online sentence-picture processing are consistent with theories of perceptual simulation.

## Introduction

Our daily lives are made up of a series of events or situations, each having a specific temporal structure. Brushing your teeth, driving to work, winking at your daughter–some events take longer than others, and there are some that are finished as soon as they begin. Although each event has its own inherent temporal structure, language cues can influence our temporal perspective on events in the theater of our minds, drawing short events out longer than usual, or collapsing long events into a flash. Verb aspect is the grammatical construction that denotes an event’s duration, onset, and completion status, and the current study was designed to investigate how this grammatical cue constrains our understanding of events.

### Temporal event structure

A critical distinction in the temporal structure of events is that between the perfect and imperfective aspect. Linguistic theory explains that the perfect aspect (1) does not allow access to the internal structure of an event, but rather treats the event as a closed unit. In this way, the beginning, middle and endpoint of the event are collapsed into an indivisible whole, and the focus is on the resulting state.

1. John had opened the bottle.
2. John was opening the bottle.

Mental representations associated with perfect sentences such as (1) should contain information about the state of affairs and highlight the consequences of the described action. Alternatively, the imperfective aspect (2) allows access to the internal structure of an ongoing event. This allows the comprehender to take a within-the-event perspective in such a way that the onset has already occurred, but the endpoint has not yet been reached [1, 2, 3, 4]. Mental representations triggered during the understanding of imperfective sentences such as (2) would focus on the action and its constraints, providing information about what is being done and how.

Previous research has demonstrated that comprehenders indeed take grammatical aspect into account when comprehending described events. For instance, Madden and Zwaan [5], Magliano and Schleich [6], Mozuraitis and colleagues [7], and Morrow [8] all found that the use of the imperfective aspect yields a representation of the situation that is not yet completed, whereas the perfective or perfect aspect yields a representation of the completed situation. Perhaps consequentially, solution rates in a problem solving task are better when the imperfective rather than perfective aspect was used, but only when the solution depended on the way an action was being done. For problems that did not focus on the action, solution rates were better when the perfective aspect was used [9].

In addition, various studies have demonstrated how verb aspect can influence the availability of described situations and their features. Characters in short narratives are subsequently more accessible when their actions are described in the imperfective rather than the perfect aspect [10, 11]. Likewise, typical locations of situations are primed by imperfective verbs, but not by perfect verbs [12]. Typical instruments used in situations are more available when situation descriptions make use of the imperfective rather than the perfective aspect [13]. Imperfective descriptions also enhanced accessibility of intention-related concepts and led to greater intentional attributions than perfective descriptions [14]. Even the overall activation level of a situation over time is greater for situations described in the imperfective rather than the perfective aspect [6]. Becker, Ferretti and Madden [15], found that reintroductions of target events later in a story were easier to integrate when the target event had been initially described in the imperfective (event left ongoing) rather than the perfective aspect (event described as finished). Describing past actions in the imperfective rather than the perfective aspect has been shown to promote memory for action-relevant knowledge and reenactment of these actions in a future context [16]. In line with such findings, Coll-Florit and Gennari [17] found that imperfective verbs took more time to read and were related to a larger range of semantic associations than perfective verbs, suggesting that events described in the imperfective aspect involve more extensive semantic processing.

Recent studies have also shown that the influence of grammatical aspect on cognitive processing goes beyond methodologies targeting overt language use. For instance, Athanasopoulos and Bylund, [18] investigated the effects of aspect on non-verbal categorization, demonstrating how not only verbalizations but also non-verbal memories for video clips are more endpoint focused in Swedish than in English participants, because the Swedish language does not allow for a progressive (ongoing) verbalization. In addition, Flecken and colleagues [19] demonstrated an effect of grammatical verb aspect on the P3 wave in event-related brain potentials. German-speaking participants showed larger P3 amplitudes for pictures that matched the endpoint rather than the trajectory of preceding motion videos. English-speaking participants did not show this difference because English is an aspectual language that focuses trajectory and endpoint equally, whereas German verbalization focuses the endpoint rather than the trajectory. This highlights how a nonlinguistic task such as motion perception is sensitive to attentional biases instantiated by the grammar of a language.

While significant progress has been made in our understanding of how verb aspect affects the representations of described information, several issues remain to be explored. In particular, the focus of the event in perfect(ive) sentences is unclear. The imperfective aspect seems to boost availability of features of the event as well as the event as a whole, but does this mean that events described in the perfect(ive) aspect are somehow less available in our situation model? One alternative to this idea is that the perfect aspect focuses the representation on a specific facet of the event that has been under accessed in previous research. This idea will be further explored below, after the temporal nature of language representations is discussed.

### Perceptual simulation

A growing body of recent research supports the idea that comprehenders understand language by activating simulations that incorporate perceptual information [20, 21]. For instance, Zwaan and colleagues have demonstrated that comprehenders are faster to verify pictures and sentences when the perceptual constraints of the sentence match the visually displayed features, such as shape [22,23] and orientation of objects [24], as well as direction of movement [25]. According to the simulation view of language comprehension, the words in a phrase or sentence activate lexical level simulations (general word meanings) that are combined to yield situation-specific simulations of the larger phrase or sentence.

These situation simulations are partial reactivations of traces of our experience, and therefore, they necessarily imply a spatio-temporal perspective on the described situation. Thus, when reading about walking in the rain or writing a letter, comprehenders simulate the experience of the described situation, including temporal and spatial features of the objects and instruments in the situation. In this case, readers’ representations should be more likely to include instruments and objects simulated in use (open umbrella, uncapped pen) rather than instruments that are not in use (closed umbrella, capped pen). The grammar of a sentence is an important way to constrain the simulation, and therefore grammar should have measurable effects on how the described situation is simulated. When the perspectives and constraints of a presented image overlap with those of the perceptual simulation activated by a sentence, then this overlap should produce processing facilitation.

Comprehenders do in fact seem to use verb aspect as a cue to regulate the activation of ongoing simulations of situations over time. Madden & Therriault [26] measured word-by-word reading as well as sensibility judgments on sentences in which a target word had been replaced by a picture (*John was working/had worked on his* *laptop* *in the study*). For the past imperfective sentences, participants were faster to process the picture (laptop), the two words following the picture, and the sensibility judgments when target entities were pictured in use (open laptop) rather than not in use (closed laptop). However, this in-use facilitation was limited to processing of the picture for the past perfect sentences. The two words following the picture, as well as the sensibility judgment were no faster when entities were pictured in use rather than not in use for these past perfect sentences.

Although it is clear that perceptual simulations are activated during language comprehension, and that grammar serves to constrain these simulations, it is not well understood how this process unfolds in real-time. As mentioned above, it remains unclear what part of a simulation is focused in the case of past perfect sentences. Madden & Therriault [26] did not specify what role the target entity played in the event. It was never the agent, but it could be an instrument used to perform an action, a resulting object of the action, or any other entity that participated in the action. Because the perfect aspect focuses the resulting state of a situation [1], it is perhaps the resulting object that is focused in the case of the perfect aspect (completed action).

### The present study

To investigate how grammatical aspect constrains event simulations at specific event roles during online sentence comprehension, we used a rebus sentence paradigm similar to that of Madden & Therriault [26], but we replaced two target words with images rather than just one word replacement. We asked participants to read imperfective (John *was using* a corkscrew to open the bottle in the restaurant) and perfect (John *had used* a corkscrew to open the bottle in the restaurant) event descriptions, word-by-word, with the critical instrument word replaced by a picture of that instrument in-use or not in-use, and the critical object word replaced by a picture of the object in its ongoing or resultant state. Response times to process the two pictures in the sentence were measured, as well as accuracy and response times of sensibility judgments to the sentences.

Before proceeding to a detailed description of the methods, we first provide the motivation for our grammatical choices in testing the aspectual distinction. Furthermore, as the experimental sentences were presented in French to native French speakers, a brief discussion of the aspectual system in French is also warranted. For various reasons (and in line with the majority of research on verb aspect), we chose to focus on the past tense. First, much of our narrative descriptions are in the past tense, as we usually re-tell our experiences after they have happened. Second, as the present can be considered an infinitely small point in time, the present tense does not offer a suitable testing ground for the aspect comparisons. This is especially the case in English, with simple present verbs often being coerced to iterative and play-by-play interpretations (he goes to school, he steps up to the plate), and present perfect verbs often being used to evoke interpretations of ongoing, repeated, or past events (I haven’t seen him (yet today… I’m still looking); She has babysat for that family (ten times/since 1982… and she still does)). Finally, the past tense provides a better alignment of tense-aspect systems in English and French.

While English and French aspectual systems differ substantially in the present tense, the English imperfective and past perfect find quite similar counterparts in the French past tense. In terms of imperfect options in the past tense, the French imparfait (utilisait) corresponds almost exactly to the English imperfective (was using). However, contrary to the constrained English tense-aspect system, French offers various ways to express the perfect in the past tense, such as the passé composé, the plus-que-parfait, and the passé simple. In all cases, the entire situation is described at once (the beginning, middle, and especially the end of the event are included in the scope of the description). The passé simple (utilisa) invokes a point of reference that is in the past and corresponds to the entire interval of the described event, whereas the passé composé (a utilisé) and the plus-que-parfait (avait utilisé) both take a point of reference that is later than the described event, providing a focus on the resulting state. Because this focus on the resulting state is in line with the aims of the present study, the latter two composed forms were considered preferable to the passé simple (which has also entirely disappeared from French spoken discourse and is only used in written literature). The plus-que-parfait was chosen over the passé composé because the point of reference is before (rather than coinciding with) the moment of the speech act, making the plus-que-parfait most comparable to the past perfect English counterpart in our previous work (had used) as well as the French imperfect form chosen for the present study (imparfait).

We expect that grammatical aspect will modulate the stage of the simulated event, such that imperfective sentences (was using) elicit ongoing event simulations as well as shorter response times for in-use/ongoing pictures than not-in-use/finished pictures, and perfect sentences (had used) elicit finished event simulations as well as longer response times for in-use/ongoing pictures than not-in-use/finished pictures. In addition, the perfect aspect is thought to focus the resultant state of events, whereas the imperfective aspect instead focuses agents, instruments, locations, etc., of events. Therefore, we expect that there would be a greater focus (and larger congruence effect) on resulting patients/objects for perfect sentences than for imperfective sentences, and perhaps a greater focus (and larger congruence effect) on instruments for imperfective sentences. However, objects may also be in focus for the imperfective aspect because it is more inclusive of any object or entity relevant to the ongoing action (resulting object included). In order to control for context effects due to sentence position of instruments and resulting objects, two experiments were run with the instrument appearing first in Experiment 1 (John *was using/had used* a corkscrew to open the bottle in the restaurant.), and the resulting object appearing first in Experiment 2 (John *was opening/had opened* the bottle with a corkscrew in the restaurant.)

## Experiment 1 Materials and methods

### Experiment 1 Participants

Twenty-eight native French-speaking volunteers, with normal or corrected-to-normal vision took part in this experiment. Both this and the following experiment were conducted in agreement with the code of ethics of the world medical association declaration of Helsinki and were performed under approval (Authorization No. 10028) from the Rhône-Alpes Préfecture review board authorizing biomedical research at the Stem Cell and Brain Research Institute. Participants were informed about the experimental procedures before they provided written consent, and they received a financial compensation for their participation.

### Experiment 1 Stimuli

**Fig 1:**
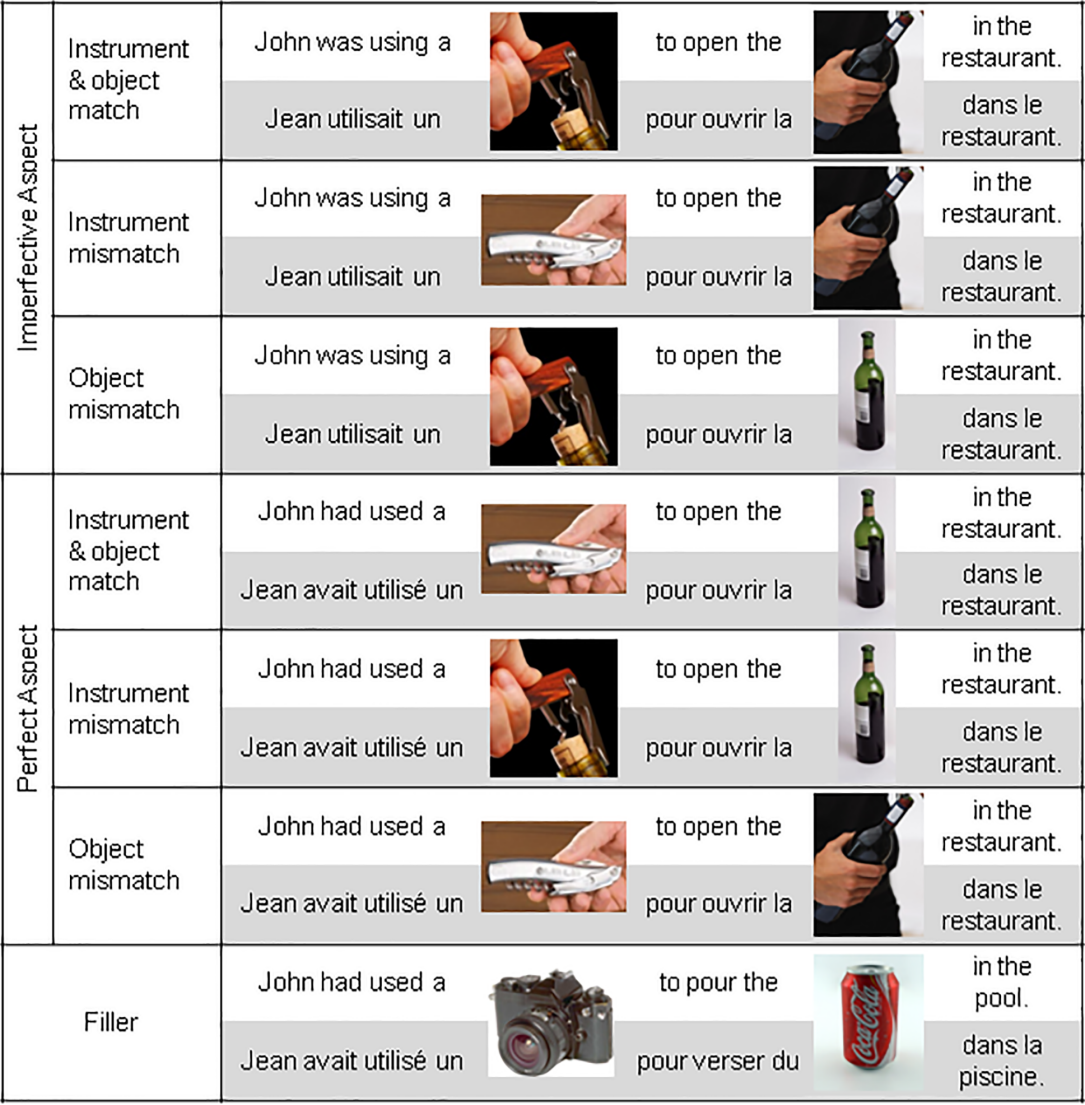
Sample stimuli used in Experiment 1with English translations.

Sixty French sentences were generated to describe a named person using an instrument or tool to act upon an object (*i*.*e*., *John was using a corkscrew to open the bottle in the restaurant*. */* In French: *Jean utilisait un tire-bouchon pour ouvrir la bouteille dans le restaurant*.). For each of the 60 sentences, 6 versions were created. First, 2 versions were created to express each sentence in the perfect aspect (*John had used… / Jean avait utilisé…*) and the imperfective aspect (*John was using… / Jean utilisait…*). Next, for both the perfect and imperfective sentence versions, the instrument word (corkscrew) was replaced by an image of that instrument either in use (corkscrew open in hand at correct orientation) or not in use (corkscrew closed). Finally, the object word (bottle) was replaced by an image of that object depicted while the action was in progress (bottle being opened) or completed (uncorked bottle) (See Fig 1 for example stimuli). Sentences always included three words after the second picture, so as not to end the sentence on a critical picture stimulus. All sentences and images described/depicted high frequency objects and actions with which participants would be familiar. In addition to the experimental stimuli, 64 similar filler sentences were generated, each with two words replaced by images, as in experimental sentences.

The 360 experimental sentences (6 versions of the 60 original sentences) were counterbalanced across 2 lists that each presented a given sentence 3 times (3 different versions) in 3 separate blocks. Each of these three blocks presented all 60 sentences (10 sentences in each of the 6 possible combinations), plus 20 of the filler sentences, resulting in 80 sentences per block and 240 trials total in the experiment. Once an entire block of 80 items (order randomized within the block) was presented, the second block started, containing the same 60 experimental items but in different condition versions (and with new first names) and 20 new fillers. Likewise for the third block. A subject only saw a given experimental item in one aspectual form, so that the three versions of that item were either ALL perfect or ALL imperfective. In either case, the three versions presented were congruent, instrument-incongruent, and object-incongruent. If an item appeared as imperfective in one list, it appeared as perfect in the other list, and vice versa. Participants either saw the first or the second list, and order of the blocks (which sentence version was presented first, second or third) was counterbalanced across participants.

Instructions for the experiment were presented on a computer screen. On a given trial, participants read a sentence that appeared on a black screen one word at a time in white font, with two of the words replaced by images. Stimuli were presented in a continuous sequence, each word disappearing and the next appearing upon a spacebar press. At the offset of the final word of the sentence, the word “Acceptable?” appeared in the center of the screen. The participants’ task was to judge whether or not the sentence made sense by pressing one of two response buttons on a keyboard (left-right answers balanced across participants). In the 60 filler sentences (20 randomly placed in each block), either the object or the instrument was replaced by an image that did not make sense with respect to the rest of the sentence context, and therefore the participant was required to press the “no” button. For all 180 experimental sentences (60 in each block), the object and instrument words were replaced by images of those objects/instruments and thus the sentences retained their meaning so that participants were required to press the “yes” button. The latency and response accuracy of these responses were recorded. The first 4 trials were practice trials, in order to familiarize participants with the mode and timing of presentation. The visual presentation and recording of responses were controlled by the E-Prime stimulus presentation software [27] on a PC.

Although the sentences always made sense on the experimental trials, two thirds of the sentences contained either an instrument or object that did not match the temporal constraints of the grammatical aspect of the verb. For instance, if the sentence states “John was using a corkscrew to open the bottle in the restaurant” then the event is ongoing, in which case in aspect-congruent trials, the matching image of the corkscrew would be in use (not closed) and the matching image of the wine bottle would be half open (not fully open). The aspect-incongruent, yet still sensible versions would either replace the corkscrew image with a corkscrew that was not pictured in use or the bottle image with a fully opened bottle. In this case, the participant would still press the “yes” button to indicate that the sentence makes sense, but this should require more processing effort and thus elicit longer response times. On these “mismatching” experimental trials, we did not expect the participants to explicitly notice this slight mismatch in temporal structure of the described events.

## Experiment 1 Results

The experiment employed a 2 (Verb Aspect: perfect/imperfective) X 2 (Picture Congruence: congruent/incongruent with the ongoing or completed nature of the verb) X 2 (Sentence Role: instrument or object picture) repeated measures design, in which all three factors varied within subjects. The ANOVA was run on response times to the images themselves as well as response times and accuracy scores for the sensibility judgments. Although list was included as a between-subjects factor in all ANOVAs, effects for the list variable are not reported, given the lack of theoretical relevance [28, 29]. Sentences producing incorrect responses were not included in the reported analyses (except for the analysis of accuracy scores on sensibility judgments), and any response time above or below 2 standard deviations from a participant’s mean for a given condition was removed prior to running the analyses. This constituted removal of 4.8% of the data. The means (and standard deviations) for the response times to images are displayed in Table 1.

**Table 1:** Means (and standard deviations) for the response times to instrument and object pictures by verb aspect.

	Instrument Picture		Resulting Object Picture	
	Congruent	Incongruent	Congruent	Incongruent
Imperfective	682 (315)	738 (463)	505 (183)	496 (211)
Perfect	664 (253)	699 (319)	484 (191)	533 (267)

The overall mixed ANOVA with list as a between subjects factor showed a main effect of Sentence Role, indicating that participants were faster to respond to object images than instrument images [F(1,22) = 24.38, p < .0001, MSe = 82332]. This is most likely due to the later placement of the object images in the sentences. Also observed was a marginally significant main effect of Congruence, suggesting that participants were faster to respond to images in general when they were congruent rather than incongruent with the temporal constraints of the preceding verb in the sentence [F(1,22) = 4.13, p = .05, MSe = 17931]. These effects were qualified by a three-way interaction among Congruence, Role, and Verb Aspect [F(1,22) = 4.84, p < .05, MSe = 7187].

In order to better understand this interaction, separate ANOVAs were conducted on the response times for imperfective and perfect sentences. The ANOVA on perfect (finished) sentences showed a main effect of Sentence Role [F(1,22) = 22.73, p < .0001, MSe = 33182], again most likely due to sentence placement of instruments and objects. More interestingly, there was a main effect of Congruence [F(1,22) = 5.72, p < .05, MSe = 8296], which was largely driven by the object images [F(1,22) = 5.17, p < .05, MSe = 6645] and not significant for the instrument images. The ANOVA on imperfective (ongoing) sentences showed the main effect of Sentence Role [F(1,22) = 20.79, p < .001, MSe = 61975], as well as a marginally significant interaction between Congruence and Role [F(1,22) = 3.95, p < .06, MSe = 12183], due to the fact that instruments showed a greater Congruence effect than objects, though neither was significant. No Congruence effects were observed on the sensibility judgments or the times to make these judgments.

The largest effect of congruence was observed on pictures of resulting objects in perfect (finished) sentences, suggesting that the perfect aspect focuses on objects more than instruments in described events. However, this effect could also arise from the fact that objects occur later in the sentence than instruments, and therefore objects are more susceptible to the effects of sentence context. Therefore, a second experiment was designed to test the idea that objects are more likely than instruments to show congruence effects even when they occur earlier in the sentence.

## Experiment 2 Materials and methods

### Experiment 2 Participants

Twenty-two native French-speaking volunteers, with normal or corrected-to-normal vision took part in this experiment. With 22 participants in our repeated-measures ANOVA design, the power to detect the match effect on resulting objects in perfect (finished) sentences at α = .05 with an effect size of .43 (as in Experiment 1) is greater than .8.

### Experiment 2 Stimuli

The sentences and pictures used in Experiment 2 were identical to that used in Experiment 1 except that the order of instruments and resulting objects was reversed so that the new sentences first mentioned the resulting object and then the instrument (*i*.*e*., *John was opening the bottle with a corkscrew in the restaurant*. */* in French: *Jean ouvrait la bouteille avec un tire-bouchon dans le restaurant*.). For some of the sentences, the three final words had to be re-written so that the sentence still made sense. Again, the purpose of the order manipulation in Experiment 2 was to verify that the strongest congruence effect observed on resulting objects in Experiment 1 was not due to the fact that the object occurred towards the end of the sentence and thus was more contextually constrained than the instrument, which occurred earlier in the sentence.

## Experiment 2 Results

The same statistical design as Experiment 1 was used in Experiment 2. The response times above or below 2 standard deviations from a participant’s mean for a given condition were again removed, constituting 5.1% of the data. The means (and standard deviations) for the response times to images are displayed in Table 2.

**Table 2:** Means (and standard deviations) for the response times to object and instrument pictures by verb aspect.

	Resulting Object Picture		Instrument Picture	
	Congruent	Incongruent	Congruent	Incongruent
Imperfective	486 (131)	477 (147)	434 (103)	444 (120)
Perfect	467 (126)	507 (145)	415 (117)	427 (108)

The overall mixed ANOVA with list as a between subjects factor showed a main effect of Sentence Role, indicating that participants were faster to respond to instrument images than object images [F(1,16) = 23.29, p < .001, MSe = 5374]. As this effect is in the opposite direction as Experiment 1, this confirms that the effect of sentence role is due to the placement of the instrument and object images in the sentences. Also observed was a marginally significant main effect of Congruence as in Experiment 1, suggesting that participants were faster to respond to images in general when they were congruent rather than incongruent with the temporal constraints of the preceding verb in the sentence [F(1,16) = 3.70, p = .07, MSe = 1439]. These effects were once again qualified by a marginally significant three-way interaction among Congruence, Role, and Verb Aspect [F(1,16) = 3.29, p < .09, MSe = 860].

As in Experiment 1, separate ANOVAs were conducted on the response times for imperfective and perfect sentences. The ANOVA on perfect (finished) sentences showed a main effect of Sentence Role [F(1,16) = 25.20, p < .001, MSe = 3467], again due to sentence placement of instruments and objects. More interestingly, there was a marginally significant main effect of Congruence [F(1,16) = 3.61, p < .08, MSe = 2850], which was largely driven by the significant effect of Congruence for object images [F(1,16) = 4.50, p < .05, MSe = 2936] and not significant for the instrument images. The ANOVA on imperfective (ongoing) sentences showed only a main effect of Sentence Role [F(1,16) = 14.41, p < .01, MSe = 2911]. No Congruence effects were observed on the sensibility judgments or the times to make these judgments.

## Discussion

In the present study, we analyzed online processing times for individual words and pictures in rebus sentences that described an agent using an instrument to perform an action on an object. We manipulated the aspect of the verb in the sentences (perfect described a completed action, *John had used the corkscrew to open the bottle in the restaurant;* and imperfective described an ongoing action, *John was using the corkscrew to open the bottle in the restaurant*). In addition, words corresponding to the instrument and object in the sentences were replaced by images depicting these objects or instruments in a way that was either congruent or incongruent with the aspect of the verb (pictured during an ongoing or completed action).

The predicted effected of congruence was indeed observed, and in both experiments this effect was driven by the resulting objects in the perfect aspect sentences. For these perfect sentences, images of objects that are depicted as having finished receiving an action evoke faster responses than objects that are depicted as currently receiving an action. Imperfective sentences tended to show an advantage for images that were congruent with the temporal phase of the preceding verb (especially for instruments), but the effect was marginal, which may be due to the less constraining temporal range of the imperfective aspect. When an event is described in the imperfective aspect, the simulation is open to various stages of the ongoing event, perhaps even including the finished state. On the other hand, the perfect aspect strongly constrains the simulation to a single temporal perspective of the event with clear expectations that the action on the recipient object be completed. Because the action is represented as a completed whole, it is the resulting object that is most likely at the focus of representations for perfect sentences [1, 2, 3, 4]. Therefore, an object pictured while receiving an action is less congruent with perfect event descriptions than an object pictured as having already received the action.

Following this same reasoning, we expect a greater role focus effect for the perfect aspect than for the imperfective aspect. The perfect aspect focuses on the resultant state of the event, and attention is highly constrained to the object that has received the action, whereas the instrument is not as relevant for the simulation. Therefore incongruent instruments are less bothersome than incongruent objects in the perfect aspect. On the other hand, the imperfective focuses on the ongoing action, and both the instrument and the recipient object are included in the simulation. This is most likely why both instruments and objects tend towards congruence effects. In both experiments the congruence effect looks to be greater for instruments than for objects in imperfective sentences, but this difference did not reach significance.

A second experiment was run in order to test whether the congruence effects observed in Experiment 1 were due to the placement of the object and instrument in the sentence. As the object occurred later in the sentence in Experiment 1, it is possible that the congruence effect observed only for objects and not for instruments was due to the buildup of context. Experiment 2 confirmed that even when the resulting object occurred before the instrument in the sentence, the congruence effects were still strongest for objects in perfect sentences. Reversing the word order in Experiment 2 also confirmed that the effect of Role was indeed an effect of sentence placement of the objects and instruments; later placement in the sentence evokes shorter processing times as context builds.

The fact that the congruence effect was strongest on objects is consistent with work on goal-directed action understanding in infants and adults. These studies demonstrate that in understanding goal-directed actions, the resulting state of the object, i.e., the goal of the action, is central and is the most important aspect of the representation of these events, much more that the instrument or means used to perform the action. Gergely, Bekkering and Király [30] have demonstrated that 14 month-old children will reproduce the goal of an action demonstrated to them by an adult, by choosing the most rational action to reach this goal, even if the adult used a non-rational way to reach the same goal. This shows that even very young children put more emphasis on the goal of an action rather than the manner of performing the action (see [31] and [32] for reviews, and [33] for treatment of this issue in adults).

From a linguistics perspective, our results are consistent with theories claiming that grammatical information such as verb aspect is used in real-time to constrain the mental simulation of meaning. In perfect sentences (completed: *had opened*), the object is focused whereas the instrument seems to be less relevant (no differences for congruent vs. incongruent pictures). This suggests that indeed the perfect aspect does not focus the representation of detailed parts of the action such as the instrument, thereby leading to the absence of aspect congruence effects on the instrument.

These results are compatible with previous data on verb aspect. Madden and Zwaan [5] presented sentences either in the perfective (“The boy built a doghouse.”) or imperfective aspect (“The boy was building a doghouse.”), followed by a two-picture choice. Their design investigated stage of the event (ongoing, completed) rather than roles in the event (agent, instrument, object), but the constraining feature of the perfective aspect is consistent with the current results. Participants in Madden and Zwaan’s [5] study were more likely to choose the completed picture after having read a perfective sentence, but chose the in-progress (half-built doghouse) and completed picture (finished doghouse) equally often after having read the imperfective sentences. In a second experiment, responses to verify that the picture matched the sentence were faster for the completed picture rather than the in-progress picture after having read a perfective sentence. There was no difference in response speed for the two picture versions after having read an imperfective sentence. Experiment 3 reversed the order of presentation, so that one of the picture versions was presented first, and then participants were asked to judge whether a subsequent sentence matched the depicted situation. Once again, only on the perfective sentences were participants faster to respond if they had previously viewed the completed pictures than if they had viewed the in-progress pictures. Participants responded to imperfective sentences equally fast whether they had viewed the in-progress or completed pictures.

In that study, all three experiments showed that participants preferred pictures showing completed situations to pictures showing ongoing situations upon reading perfective sentences but showed no picture preference upon reading imperfective sentences. This apparent lack of constraint in the imperfective construction does not necessarily mean that participants were not activating ongoing representations of the imperfective sentences. Rather, this lack of effect was hypothesized to arise from the fact that ongoing situations may be represented in various stages of completion, even approximating the endpoint of the situation. Therefore, it is more difficult to capture the appropriate stage of completion that would effectively match the participants’ representations of the imperfective sentences in the picture stimuli. Conversely, the endpoint of a situation is temporally well-specified, and thus it is easier to pictorially capture the appropriate stage of completion that would effectively match the participants’ representations of the perfective sentences.

In conclusion, the present study adds to an expanding body of research showing how grammatical features of verbs can have important consequences for how events are processed and represented. The temporal structure of events is a very basic part of our experience, so it is natural that this structure must also be specified in our representation of events cued through language. In addition, this study provides novel insights on how grammar constrains the focus on various roles in events, with a strong focus on resulting object in the case of the perfect aspect. Learning how grammar constrains our simulations of events is crucial to understanding how people activate meaningful mental representations of situations during language comprehension. Thus, it is important to continue this line of research on verb grammar to further identify temporal-semantic features that constrain language simulations.

## Supporting information

S1 Dataset Experiment 1 averaged and individual data tables. (XLSM)

S2 Dataset Experiment 2 averaged and individual data tables. (XLSM)
